# High genetic carrier frequency of Wilson’s disease in France: discrepancies with clinical prevalence

**DOI:** 10.1186/s12881-018-0660-3

**Published:** 2018-08-10

**Authors:** Corinne Collet, Jean-Louis Laplanche, Justine Page, Hélène Morel, France Woimant, Aurélia Poujois

**Affiliations:** 1 Department of Biochemistry and Molecular Biology, Lariboisiere University Hospital, APHP, 2 rue Ambroise Paré, 75010 Paris, France; 2 INSERM U1132, University Paris-Diderot and Department of Rheumatology, Lariboisiere University Hospital, Paris, France; 3 National reference Centre for Wilson’s Disease (CRMR Wilson), Department of Neurology, Lariboisiere University Hospital, APHP, Paris, France

**Keywords:** Wilson’s disease, *ATP7B*, Clinical prevalence, Heterozygous carrier frequency, Epidemiology, Copper

## Abstract

**Background:**

Wilson’s disease (WD) is a rare autosomal recessive metabolic disease caused by *ATP7B* gene mutations tat cause excessively high copper levels, particularly in the liver and brain. The WD phenotype varies in terms of its clinical presentation and intensity. Diagnosing this metabolic disorder is important as a lifelong treatment, based on the use of copper chelating agents or zinc salts, is more effective if it’s started early. Worldwide prevalence of WD is variable, with an average of 1/30,000. In France, a recent study based on French health insurance data estimated the clinical prevalence of the disease to be around 3/200,000.

**Methods:**

To estimate the genetic prevalence of WD in France, we analysed the *ATP7B* gene by Next Generation Sequencing from a large French cohort of indiscriminate subjects.

**Results:**

We observed a high heterozygous carrier frequency of *ATP7B* in France. Among the 697 subjects studied, 18 variants classified as pathogenic or probably pathogenic were found at heterozygous level in 22 subjects (22 alleles/1394 alleles), yielding a prevalence of 0.032 or 1/31 subjects.

**Conclusions:**

This considerable and unexplained discrepancy between the heterozygous carrier frequency and the clinical prevalence of WD may be explained by the clinical variability, the incomplete penetrance and the existence of modifiers genes. It suggests that the molecular analysis of *ATP7B* should be interpreted with caution, always alongside copper assays (ceruloplasmin, relative exchangeable copper, 24 h-urinary copper excretion) with particular respect to exome sequencing.

## Background

Wilson’s disease (WD) is a rare autosomal recessive disorder caused by mutations to the *ATP7B* gene (13q14.3). *ATP7B* encodes a member of the P-type cation transport ATPase family that has functions in exporting copper out of cells, such as the efflux of hepatic copper into bile. WD is an inherited metabolic disorder which results in excess copper levels, particularly in the liver and brain. The initial symptoms may vary; in more than half of patients they may include hepatic abnormalities such as chronic active hepatitis, cirrhosis and more rarely fulminant hepatic failure, whereas others might display different neuropsychiatric manifestations such as tremor, Parkinsonism, dystonia or psychiatric abnormalities [1, 2]. The clinical signs of WD can appear at different ages (usually between 8 and 30 years) but they may also occur at extreme ages (1 to 80 years) [3, 4]. The outcome of WD is generally fatal without treatment based on copper chelating agents (such as trientine and D-penicillamine), zinc salts or a liver graft in the most severe hepatic cases.

Early studies had indicated the prevalence of WD as being around 30 per one million [5], and based on the Hardy-Weinberg equilibrium, a heterozygous carrier frequency of about 1/90. Subsequent epidemiological studies have suggested a lower clinical prevalence of WD in European countries with a low degree of consanguinity, estimated at between 12 and 20 per one million [6]. Nevertheless, genetic prevalence appears to be higher than clinical prevalence, as shown by two recent British and Korean studies which produced heterozygous carrier frequencies of 1/25 and 1/53, respectively [7, 8].

In France, WD patients are followed by the National Reference Centre for Wilson’s disease. According to the French National Health Insurance Information System database, the clinical prevalence of WD is around 3/200,000 [9]. In order to determine the heterozygous carrier frequency of *ATP7B* in France, we studied a large cohort in non-WD individuals by sequencing the entire coding regions of *ATP7B* associated with its 5’UTR regions and promoter.

## Methods

### NGS sequencing and genetic analysis

Our retrospective study included anonymised data on 697 individuals between January 2015 and May 2017. These patients who had no family history of WD were French residents treated in 18 French university hospitals and had various indications other than WD, hepatic or neurological diseases. After automated DNA extraction (Qiagen, Courtaboeuf, France), the DNA samples were screened by NGS using a panel of various genes which included *ATP7B*. Library preparations for NGS were established using the surelectQXT kit (Agilent, Les Ulis, France) based on a hybrid capture system for sequencing on the Miseq sequencer (Illumina, Paris, France). The bioinformatics pipeline included the miseq-reporter followed by Fastq alignments with SeqNext and visual variant control (JSI Medical Systems, Ettenheim, Germany). The variants were included if the depth of lecture was greater than 30X with a variant frequency ranging from 0.40 to 0.60. The design included the promoter, the 5’UTR region, the 3’UTR region and the coding region of the *ATP7B* gene (10,000 pb) with Refseq for the nomenclature: *ATP7B* (NM_000053.3) for the exonic regions and intronic-exonic boundaries; *ATP7B* (NG_008806.1) for exon numbering and the intronic region. Studies of missense variant pathogenicity involved the use of predictive software programs such as Polyphen-2 (http://genetics.bwh.harvard.edu/pph2/), PhyloP (http://compgen.cshl.edu/phast/), and CADD (http://cadd.gs.washington.edu) and MutationTaster (http://www.mutationtaster.org), while splicing variant pathogenicity was determined used predictive software such as MaxEntScan (http://genes.mit.edu/burgelab/maxent), Human Splicing Finder (http://www.umd.be/HSF3/) and NNSPLICE (http://www.fruitfly.org/seq_tools/splice.html).

The frequencies of all variants were searched for in Exome Aggregation Consortium (ExAc, exac.broadinstitute.org), Exome Sequencing Project (ESP, evs.gs.washington.edu) and dbSNP (https://www.ncbi.nlm.nih.gov/projects/SNP). We retained variants with an allelic frequency lower than 0.06% and variants already described in the literature and reported in the Professional Human Gene Mutation Database (HGMD, http://www.hgmd.cf.ac.uk/ac/index.php), the Wilson Mutation Database, University of Alberta, Canada (http://www.wilsondisease.med.ualberta.ca) and in our Lariboisière Wilson’s disease Database (Paris), which includes 496 index cases.

All the variants identified were classified according to ACMG guidelines including data as Minor Allele Frequency (MAF) in the general population, results of the predictive software programs and descriptions in the literature. Variants were considered to be:

- pathogenic when they had been described in the literature without any ambiguities as to their interpretation;
- likely pathogenic when they had not been described in the literature or professional HGMD, but were classified as deleterious based on all subsequent in silico predictive algorithms and having MAF < 0.06%.
- Variant with Uncertain Significance (VUS), when is conflicting: the criteria for benign and pathogenic are contradictory by using ACMG guidelines.

## Results

In order to determine the genetic prevalence of Wilson’s disease in French population, the promoter, the 5’UTR region, the 3’UTR region, all coding region and exon-intron junctions of ATP7B gene were sequenced in 697 DNA samples. We identified 4222 variants in coding *ATP7B* across the 697 subjects. Of these, 64 variants have a MAF < 1% and were seen in 94 subjects. The average number of *ATP7B* variants with MAF below 1% identified per subject was 0.142. No Copy Number Variation was detected by NGS.

Among the 697 subjects studied, 23 heterozygous variants were classified as pathogenic, likely pathogenic and VUS (Table 1). These 23 heterozygous variants were found in 37 individuals, leading to a *ATP7B* variant carrier frequency of 0.0265 (37/1394 alleles) or a heterozygous carrier frequency of 1/19 subjects.

**Table 1:** Details of the 23 ATP7B variants retained, based on the three classifications of pathogenic, probably pathogenic and VUS

Chromosomal position/change	rs ID	Allele Count	Exon	Nucleotide	Protein	Protein Domain	Max ExAC MAF (%),	PhyloP	PP	MT	CARD	Allele Count (1394 total alleles),	Lariboisiere database allele count (1208 total alleles)	Classification	Ref
g.52549234 T > C	rs201738967	1	2	c.122A > G	p.Asn41Ser	–	European: 0.040%	2.63	D	DC	24.4	1	1	pathogenic	[14]
g.52535997A > G	rs186924074	2	6	c.1922 T > C	p.Leu641Ser	HMA	European: 0.078%	2.87	D	DC	27.4	2	1	pathogenic	[15]
g.52535985A > C	rs121907998	1	6	c.1934 T > G	p.Met645Arg	–	European: 0.021%	0.69	B	DC	14.09	1	11	pathogenic	13, [16]
g.52534281 T > C	–	1	Intron 7	c.2121 + 3A > G donor splicing site utilisation: − 100%	p.?	–	European: 0.000090%	0.69	–	DC	7.698	1	0	pathogenic	[17]
g.52532619A > G	rs760713333	1	8	c.2183A > G	p.Asn728Ser	P-Type ATPASe	Asian: 0.046%	3.43	D	DC	22.8	1	0	pathogenic	[18]
g. 52524268C > T	rs191312027	1	11	c.2605G > A	p.Gly869Arg	P-Type ATPASe	Asian: 0.023%	6.10	D	DC	34	1	1	pathogenic	[16]
g.52520559G > A	rs201061621	1	13	c.2921C > T	p.Thr974Met	P-Type ATPase	African: 0.033%	5.94	D	DC	28.8	1	0	pathogenic	11
g.52513198 T > C	rs200911496	1	17	c.3688A > G	p.Ile1230Val	P-Type ATPase	Asian: 0.012%	4.89	D	DC	24.9	1	1	pathogenic	11
g.52511409 T > C	rs565970531	1	Intron 19	c.4021 + 3A > G donor splicing site utilisation: − 100%	p.?	–	Asian: 0.2791%	1.17	–	DC	9.707	1	0	pathogenic	[19]
g.52509155G > A	rs181250704	4	21	c.4135C > T	p.Pro1379Ser	–	European: 0.18%	2.87	D	DC	28.5	4	0	pathogenic	[15]
g.52518403-52518403delinsG	–	1	14	c.3083-3085delinsG	p.Lys1028Serfs*40	P-Type ATPase	–	4.97 1.34 4.89	D	DC	–	1	1	pathogenic	this study
g.52524498C > G	rs181388674	1	10	c.2485G > C	p.Asp829His	P-Type ATPase	–	6.10	D	DC	32	1	0	Likely pathogenic	this study
g.52511803 T > C	–	1	18	c.3712A > G	p.Lys1238Glu	P-Type ATPase	–	4.81	D	DC	27.5	1	0	Likely pathogenic	this study
g.52511700G > C	–	1	18	c.3815C > G	p.Ser1272Cys	P-Type ATPase	–	5.86	D	DC	25.9	1	0	Likely pathogenic	this study
g.52534283G > A	rs751235573	1	Intron 7	c.2121 + 1G > A donor splicing site utilisation: −100%	p.?	–	European: 0.00090%	5.86	–	DC	25.6	1	0	Likely pathogenic	this study
g.52532611C > A	_	1	8	c.2191G > T	p.Val731Leu	P-Type ATPase	European: 0.0018%	6.02	D	DC	25.8	1	0	Likely pathogenic	this study
g.52513215C > A	rs532177115	1	17	c.3671G > T	p.Arg1224Leu	P-Type ATPase	African: 0.0102%	4.24	D	DC	34	1	0	Likely pathogenic	this study
g.52511739C > T	–	1	18	c.3776G > A	p.Gly1259Glu	P-Type ATPase	European: 0.0018%	3.11	D	DC	26.9	1	0	Likely pathogenic	this study
g.52508989G > A	rs60986317	9	21	c.4301C > T	p.Thr1434Met	–	African: 0.57%	1.09	D	PM	24,1	9	0	VUS	10
g.52542680A > G	rs138427376	1	4	c.1607 T > C	p.Val536Ala	HMA	Finland: 1.15%	0.45	B	PM	9.214	1	0	VUS	7, 11
g.52542666C > T	rs187046823	1	4	c.1621G > A	p.Glu541Lys	HMA	European: 0.019%	0.53	B	PM	5.930	1	0	VUS	7
g.52534410C > T	rs72552259	3	7	c.1995G > A	p.Met665Ile	–	European: 0.1685%	2.38	B	DC	24	3	0	VUS	7, [12]
g.52511626C > T	rs148399850	1	18	c.3889G > A	p.Val1297Ile	P-Type ATPase	Asian: 1.5%	1.13	B	DC	18.36	1	0	VUS	10, [13]

*PP* Polypen-2: D for Damaging, B for Benign. *MT* MutationTaster DC for Disease Causing, PM for Polymorphism HMA Heavy metal associated domain, copper ion-binding. Ref: described in Human Gene Mutation Database (HGMD)

In a second step and to be more stringent, we excluded five variants reported in the literature on WD and that we have classified as VUS according to ACMG guidelines [10–12]. The c.4301C > T (p.Thr1434Met) variant was predicted to be “probably damaging” according to Polyphen-2, but was found in nine subjects which produced a variant frequency of 0.00645 (9/1394 alleles) in our study, and moreover was never found in our large WD database. Five other subjects harboured four VUS (p.Val536Ala, p.Glu541Lys, p.Met665Ile, p.Val1297Ile) that were predicted to be benign by predictive softwares and were not present in our WD database. Moreover, the p.Val536Ala variant (c.1607C > T) (with a MAF = T at 0.45% in the European population) reached a high frequency (1.29%) in the Finnish population according to Exac. In addition p.Val1297Ile (c.3889G > A) displayed a high MAF = A at 1.5% in the Asian population [10, 13] (Table 1). Consequently, *ATP7B* heterozygous carrier frequency was recalculated to include only heterozygous variants classified as pathogenic [14–19] and likely pathogenic. Eighteen heterozygous mutations were thus found in 22 subjects, yielding a variant frequency of 0.01578 (22/1394 alleles) or a heterozygous carrier frequency of 1/31 subjects (Table 1). All these mutations were deleterious according to the predictive softwares. At a more detailed level, ten missense or splicing mutations were identified in 14 subjects, all of whom were present in HGMD and in our WD database. Among the eight other subjects**,** one novel deletion, p.(Lys1028Serfs*40) causing a frameshift mutation in exon 14, one splicing variant in intron 7, and six probably pathogenic missense variants were identified. Of the six missense variants, three are novel (p.Asp829His p.Lys1238Glu, p.Ser1272Cys) and three have a MAF < 0.011% (p.Val731Leu, p.Arg1224Leu, p.Gly1259Glu) (Table 1).

## Discussion

Our study therefore confirmed the high heterozygous carrier frequency of *ATP7B* in the French population with one in 31 subjects harbouring a pathogenic variant at a heterozygous level. Our NGS approach was similar to that used by Coffey et al. [7] and Jang et al. [8]. We only included variants with probably pathogenic or pathogenic deleterious effects. Consequently, we were able to conclude that the heterozygous carrier frequency of *ATP7B* in our French cohort (1/31) was higher than described in Korean (1/53) population and slightly lower than observed in the British population (1/25).

When based on the VUS parameter, the heterozygous prevalence of *ATP7B* (1/19) was found to be similar than that described in the British population (1/18) [8]. Among the five VUS described during our study (p.Val536Ala, p.Glu541Lys, p.Met665Ile, p.Val1297Ile and p.Thr1434Met), three (p.Val536Ala, p.Glu541Lys, p.Met665Ile) had been reported in Coffey’s publication as being benign. However, unlike our British colleagues, we considered that one of the VUS (p.Met645Arg) was pathogenic. Indeed, the frequency of this mutation was 1.8% (11/604 patients) in the Lariboisière WD cohort, and various publications have reported this mutation in the literature since the 1990s [12, 16]. Furthermore, this is the most frequent mutation reported in Spanish WD patients [20].

p.Thr1434Met and p.Val1297Ile were not found during Coffey’s study. p.Val1297Ile displays a high frequency in the Asian population (1.5%), so it would be preferable to consider it as VUS even though it has already been described in the literature [11, 13]. Also, p.Thr1434Met should be definitively reclassified as benign, despite the first Turkish publication 18 years ago [10]. Indeed, its variant frequency was elevated in our study (0.645%) and in dbSNP databases, its frequency reaching 4% in the African population. Moreover, neither variant was ever found among the 604 WD patients in the Lariboisière WD database.

The high heterozygous prevalence of *ATP7B* observed in France raises the question of the approach to be adopted in the event of a genetic WD finding. Firstly, account needs to be taken of the discrepancies between the clinical and genetic frequencies seen both in France [9] and worldwide [7, 8], for different reasons. The diagnosis is certainly underestimated, or the pathology may be misdiagnosed. Indeed, in fulminant liver or tardive forms, or with a mild neurological presentation, a diagnosis of WD may not be suggested and the cause of death incorrectly determined. In addition, asymptomatic forms may be due to incomplete gene penetrance or the presence of disease-modifying genes (*COMMD1, ATOX1, XIAP, HFE,* prion protein, methylenetetrahydrofolate MTHF reductase, apolipoprotein E) [21].

Therefore, in order to determine the clinical status of WD in cases of genetic WD, a precise exploration of the copper balance (ceruloplasmin, relative exchangeable copper and 24 h urinary copper excretion) needs to be performed and patients monitored carefully so as to determine the clinical course of an asymptomatic form [22].

Furthermore, because the heterozygous carrier frequency of the *ATP7B* mutation is higher than previously thought, the guidelines concerning familial screening need to be revaluated in the near future. The families of WD patients should more frequently be offered not only a copper balance evaluation but sequencing of the whole coding region of *ATP7B* in order to improve genetic counselling [23].

Our study highlighted the difficulties of interpreting genetic results, particularly in the case of Exome sequencing analysis. Indeed, the *ATP7B* gene is on the list of genes that should be reported as incidental or secondary findings according to the recommendations of American College of Medical Genetics and Genomics (ACMG), notably because treatments for WD are available and effective when administered prior to the onset of symptoms [24].

## Conclusions

Our study highlighted the major difference between the high heterozygous carrier frequency and the low clinical prevalence of WD. This discrepancy may be explained by the clinical variability, the incomplete penetrance and the existence of modifiers genes. Also, the molecular analysis of *ATP7B* should be interpreted with caution, always alongside copper assays (ceruloplasmin, relative exchangeable copper, 24 h-urinary copper excretion) with particular respect to exome sequencing. The variability in the major phenotype, the potential for reduced penetrance and genetic prevalence combine to hamper the diagnosis of WD. However, the existence of effective therapies urges us to detect a maximum of individuals with genetic WD before any symptoms appear.
